# Fluence Rate Differences in Photodynamic Therapy Efficacy and Activation of Epidermal Growth Factor Receptor after Treatment of the Tumor-Involved Murine Thoracic Cavity

**DOI:** 10.3390/ijms17010101

**Published:** 2016-01-14

**Authors:** Craig E. Grossman, Shirron L. Carter, Julie Czupryna, Le Wang, Mary E. Putt, Theresa M. Busch

**Affiliations:** 1 Department of Radiation Oncology, Perelman School of Medicine, University of Pennsylvania, Philadelphia, PA 19104, USA; craig_grossman@urmc.rochester.edu (C.E.G.); poseysl@mail.med.upenn.edu (S.L.C.); 2 Department of Radiology, Perelman School of Medicine, University of Pennsylvania, Philadelphia, PA 19104, USA; julie.czupryna@perkinelmer.com; 3 Department of Biostatistics, Perelman School of Medicine, University of Pennsylvania, Philadelphia, PA 19104, USA; wangle@mail.med.upenn.edu (L.W.); mputt@mail.med.upenn.edu (M.E.P.)

**Keywords:** photodynamic therapy, fluence rate, lung, HPPH, epidermal growth factor receptor, optical imaging, proliferation, thoracic cavity, non-small cell lung carcinoma

## Abstract

Photodynamic therapy (PDT) of the thoracic cavity can be performed in conjunction with surgery to treat cancers of the lung and its pleura. However, illumination of the cavity results in tissue exposure to a broad range of fluence rates. In a murine model of intrathoracic PDT, we studied the efficacy of 2-(1-hexyloxyethyl)-2-devinyl pyropheophorbide-a (HPPH; Photochlor^®^)-mediated PDT in reducing the burden of non-small cell lung cancer for treatments performed at different incident fluence rates (75 *versus* 150 mW/cm). To better understand a role for growth factor signaling in disease progression after intrathoracic PDT, the expression and activation of epidermal growth factor receptor (EGFR) was evaluated in areas of post-treatment proliferation. The low fluence rate of 75 mW/cm produced the largest reductions in tumor burden. Bioluminescent imaging and histological staining for cell proliferation (anti-Ki-67) identified areas of disease progression at both fluence rates after PDT. However, increased EGFR activation in proliferative areas was detected only after treatment at the higher fluence rate of 150 mW/cm. These data suggest that fluence rate may affect the activation of survival factors, such as EGFR, and weaker activation at lower fluence rate could contribute to a smaller tumor burden after PDT at 75 mW/cm.

## 1. Introduction

When combined with surgery, photodynamic therapy (PDT) of tumors can provide a means to eradicate residual disease that is unresectable for reasons that may include a lack of detectability (*i.e.*, microscopic disease), broad area of superficial involvement, or localization adjacent to vital structure. Clinical trials have evaluated intraoperative PDT in numerous settings that involve light delivery to a resection cavity. One example includes cavitory PDT after resection of malignant brain tumors [1]. This approach has also been extended to even more complex geometries, such as PDT of the peritoneal cavity or the thoracic cavity after surgical removal of gross macroscopic disease [2,3,4].

The intraoperative application of PDT to a surgical cavity involves significant technical challenges in delivering light to an irregular surface. In particular, complex surfaces, such as the thoracic or peritoneal cavities, can be challenging to illuminate. The delivery of PDT to any irregular surface is associated with variability in the fluence rate of illumination incident across the treated tissue [5]. Moreover, the adaption of light delivery techniques for large surface areas can introduce further spatial and temporal heterogeneities in fluence rate. For example, at our institution, a light source is systemically moved throughout the intralipid-filled thoracic or peritoneal cavity in order to deliver PDT to serosal malignancies [6]. As a result, the disease-laden tissue is exposed to a range of fluence rates over the course of treatment [7].

Different biological effects can be expected from treating at different fluence rates. Lower fluence rates are generally associated with less hypoxia during PDT and can produce more treatment-related cytotoxicity [8,9,10]. PDT-induced inflammatory response can be more pronounced at sub-curative doses of low fluence rate [11]. Moreover, as found with other cancer therapies, therapeutic response to PDT will depend upon its effect on survival signaling in tumor and tumor-associated cells [12,13]. Little is known about how or whether fluence rate plays a part in this process. PDT-induced signal transduction involves the well-studied survival pathways of mitogen-activated protein kinases/extracellular signal-regulated kinases (MAPK/ERK) and phosphatidylinositol-3-kinase/protein kinase B (PI3K/AKT), and treatment can alter the expression of growth factors and/or growth factor receptors that activate these pathways [14,15,16,17,18]. Informingly, combinational approaches that inhibit survival signaling in conjunction with PDT will benefit treatment responses, and toward this purpose there has been much interest in the study of drugs that target the epidermal growth factor receptor (EGFR) [19,20,21,22,23]. Given the roles of fluence rate in PDT-induced tissue hypoxia, oxidative stress, vascular damage, cytokine production, and other factors that can contribute to the activation of survival signaling [9,11,13], it is of interest to determine whether the induction of signaling is also a function of fluence rate.

In the studies reported here, we evaluated fluence rate effect on EGFR activation in tumor nodules of the PDT-treated murine thoracic cavity. Human tumor xenografts of non-small cell lung carcinoma (NSCLC) were grown as disseminated disease in the murine thoracic cavity [24]. In contrast to a single nodule of disease at either a subcutaneous or orthotopic site, our intrathoracic model recapitulates the multinodular and diffuse spread of pleural malignancies that are treated in clinical trials of pleural PDT. As in our clinical trials, illumination of the mice involves light exposure to the entire thoracic cavity; thus, the illumination geometry of our preclinical model could also capture clinically-relevant aspects of intrathoracic light delivery that exacerbate fluence rate effects. Results find the lower (75 mW/cm) of the tested fluence rates to be most effective in reducing intrathoracic tumor burden. In contrast to that found after intrathoracic PDT at 150 mW/cm, illumination at 75 mW/cm did not increase EGFR activation in the proliferating areas of tumor nodules with an incomplete response to PDT.

## 2. Results and Discussion

### 2.1. Fluence Rate Effects on Efficacy of Thoracic Photodynamic Therapy (PDT)

**Figure 1:**
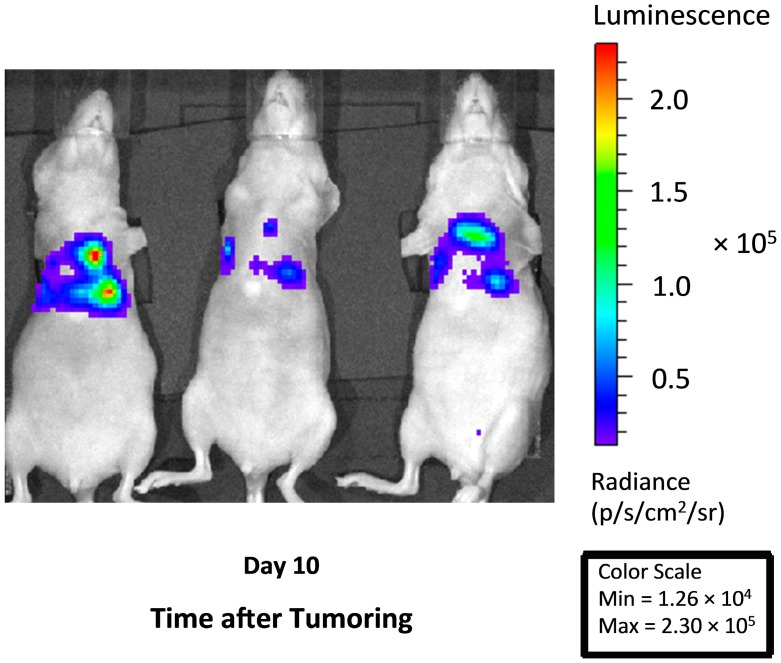
*In vivo* bioluminescent imaging demonstrates thoracic tumor burden in representative animals. Images taken at 10 days after the injection of H460 tumor cells and illustrates the thoracic spread of disease that includes areas of focal growth.

Fluence rate effects of intrathoracic PDT were investigated in our previously described murine model [24]. Disease was propagated by the percutaneous injection of H460 cells in the thoracic cavity and developed as disseminated bilateral intrathoracic masses that visibly ranged from pinpoint to mm-sized nodules. Studies employed a cell line that was transfected with luciferase and red fluorescent protein (RFP). The luciferase enzyme facilitated *in vivo* imaging via bioluminescence to identify nodules of disease (Figure 1). RFP-generated fluorescence was utilized in histologic studies in order to confirm the presence of tumor in tissue sections under analysis.

The goal of this study was to investigate EGFR activation in nodules of NSCLC that were exposed to PDT of the thoracic cavity. The expression of survival factors, such as EGFR, is of great clinical relevance in tumors that incompletely respond to treatment, as this will contribute to disease progression. We accordingly focused the present investigation on thoracic tumor nodules with evidence of proliferation after PDT, because they represent disease with an incomplete response. Illumination was performed at two incident fluence rates (150 and 75 mW/cm) and light was delivered to the murine thoracic cavity by cylindrical diffusing fibers. Tissue sampling for molecular studies (described below) was performed along the periphery of the cavity to provide spatial consistency relative to light delivery.

**Figure 2:**
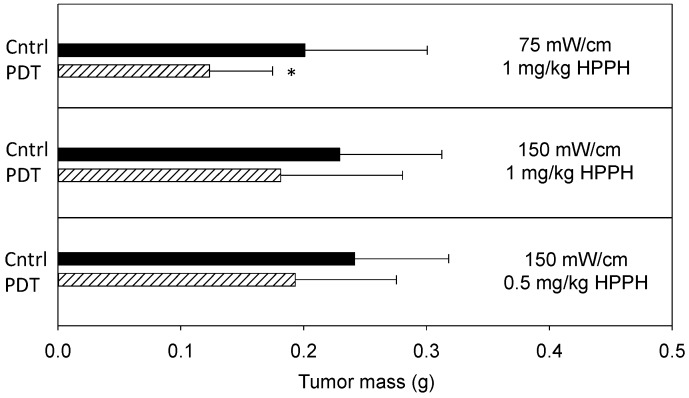
PDT of the thoracic cavity reduces total tumor mass. 2-(1-hexyloxyethyl)-2-devinyl pyropheophorbide-a (HPPH; Photochlor^®^)-PDT at the indicated fluence rates and drug doses was delivered to the tumor-bearing murine thoracic cavity to a total dose of 50 J/cm. Tumor burden was measured 4 days after PDT. Controls (Cntrl) include animals that were untreated, received illumination (but no photosensitizer) at the fluence rate corresponding to the PDT condition, or received photosensitizer (but no illumination) at the photosensitizer dose corresponding to the PDT condition. Controls behaved similarly and were therefore pooled for analysis. Plots show the mean ± SD (error bars) for each group. *n* = 8–27 animals per group. * indicates *p* < 0.05 for comparison to control.

Prior to the molecular studies, treatment efficacy was investigated to define PDT effect on disease progression at each of the two incident fluence rates (150 and 75 mW/cm). PDT consisted of the photosensitizer 2-(1-hexyloxyethyl)-2-devinyl pyropheophorbide-a (HPPH; Photochlor^®^; 1 or 0.5 mg/kg) and a total light dose of 50 J/cm. Illumination was delivered at 12 days after tumor inoculation, and treatment effect was quantified by excising and weighing tumor burden on day 16 (*i.e.*, 4 days after PDT). Figure 2 summarizes tumor burden on day 16 for treated and control conditions. Compared to controls, PDT at 1 mg/kg HPPH with 75 mW/cm produced significant (*p* = 0.02) reduction in tumor burden. Aggregate mass (mean ± SD) of control disease was 0.2009 ± 0.0998 g, whereas disease burden was 0.1246 ± 0.0538 g in animals that received PDT. In contrast, a smaller PDT effect was noted with illumination at 150 mW/cm. Treatment with 1 mg/kg HPPH at 150 mW/cm reduced tumor mass to 0.1812 ± 0.0993 g, compared to 0.2293 ± 0.0767 in controls. Because some of our previous work identified lower photosensitizer doses to be effective in pairings with higher fluence rate [25], we also tested PDT at 150 mW/cm in combination with a HPPH dose of 0.5 mg/kg. Under these conditions, PDT reduced tumor mass to 0.1931 ± 0.0826 g compared to a control mass of 0.2414 ±0.0861 g. Decreases at both 150 mW/cm conditions were detected as trends.

These studies revealed that PDT at both 75 and 150 mW/cm could reduce tumor burden, but only the decreases at 75 mW/cm were significant. These overall trends in response were also reflected in studies with the parental H460 cell line, although this line grew at a slightly slower rate and was more responsive to PDT in general. Control tumors of the parental cell line (untreated, light controls or drug controls) achieved a mass of 0.1785–0.1811 g on day 16. PDT significantly reduced tumor burden with both 75 and 150 mW/cm illumination (1 mg/kg HPPH) to 0.0861 ± 0.0487 and 0.1191 ± 0.0603 g, respectively. However, the reductions at 75 mW/cm were larger and more consistent. The underlying cause for the small shift in PDT response of the parental H460 line compared to its luciferase/RFP transfected counterpart is not known. Nevertheless, it is not uncommon for biologic differences to exist between parental cell lines and those transfected with foreign antigens, with alteration of immune responses offering one explanation [26]. In our case these effects would have been mediated through innate immune responses since tumors were propagated in nude animals (which lack T-cell mediated immunity). Due to this possibility and the fact that we previously detected an inflammatory response during intrathoracic PDT [24], we further considered PDT-induced immune cell infiltrate in the present study. Tumor-localized neutrophils were detected by staining for Gr-1, and the extent of infiltrate was quantified as a percentage of the cellular area in tumor sections. Gr-1 positive cells (mean ± SD) accounted for 11.3% ± 5.8% of tissue area in tumors exposed to 75 mW/cm and 6.5% ± 3.1% of tissue area in tumors exposed to 150 mW/cm. In control tumors (light-alone and drug-alone), Gr-1 positivity represented 5.7% ± 3.1% of the tissue. From these findings we conclude that a PDT-induced inflammatory response may contribute to tumor response after intrathoracic PDT and, as reported by others [11], we detected trends toward a stronger response at low fluence rate with sub-curative light doses. However, secondary reactions such as inflammation can be expected to occur in conjunction with primary oxidative damage from PDT, and it is the total accumulation of direct and secondary effects that mediates the PDT response. To study the sum of these effects at the molecular level, we considered the effect of intrathoracic PDT at two fluence rates on tumor EGFR expression and activation.

### 2.2. Imaging to Identify Nodules with Incomplete PDT Response

**Figure 3:**
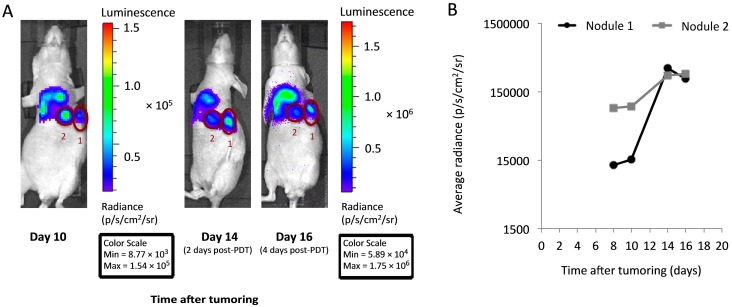
*In vivo* imaging facilitates the identification of distinct tumor nodules with an incomplete response to PDT. (**A**) Bioluminescence imaging in a representative PDT-treated mouse was used to identify well-circumscribed nodules with a minimal response to PDT (labeled as “1” and “2”). *To aid visualization, the day 10 image is not linked to other images for scaling purposes; note the differences in the values of the pre- and post-PDT scales (min/max) between the Day 10 and the Day 14 and 16 images*; (**B**) Plots of bioluminescence in each of nodules “1” and “2” demonstrate persistent signal at times after PDT (treatment at 150 mW/cm, 0.5 mg/kg HPPH). For quantification in (**B**), all images are adjusted for equivalent scale and signal is corrected for background levels.

Due to the diffuse nature of the disease under investigation, and likely heterogeneity in PDT response within this disease, we sought to identify tumor nodules with an incomplete response to PDT in which to study EGFR activation. Toward this goal, bioluminescent imaging was used for longitudinal assessment of tumor burden. Animals were imaged on days 8 and 10 after tumor cell inoculation (before PDT), continuing to days 12, 14 and 16 as post-PDT timepoints. PDT itself was performed on day 12. Distinct hotspots of nodule growth were easily visualized by bioluminescence and followed throughout the imaging time course to inform the selection of well-circumscribed disease with an incomplete PDT response. Images of this longitudinal timecourse from a representative animal are depicted in Figure 3A. Guided by these images, the nodules labeled as “1” and “2” were chosen for excision (day 16, 4 days after PDT). Figure 3B plots the *in vivo* change in nodule bioluminescent signal over time, confirming growth of the selected nodules in the days after PDT. We note here that imaging was used specifically for the purpose of selecting growing “hot spots” against a background of diffuse disease, not for the purpose of quantifying overall tumor response to PDT. If optical imaging is to be used for comparing overall PDT response between animals, it will be necessary to perform separate validation studies that test the correlation between total signal strength and total tumor burden. For the purposes of the present study, overall PDT response was assessed through the total tumor mass, as described above.

From *in vivo* imaging, we noted that PDT could promote a large increase in bioluminescent signal within the first two days after treatment. We questioned whether artifactual increases in signal from PDT-induced inflammation and hyperpermeability might occur in the absence of progressing disease. To secondarily confirm disease progression, immunohistochemistry was used to test for the presence of proliferation in the day 16 tumor nodules. Histological sections were cut from the nodules, analyzed for expression of the proliferation marker Ki-67, and the percent area of the tumor section that was positive for Ki-67 was quantified. On average, 20% ± 6% and 17% ± 13% of the tumor section was positive for Ki-67 after PDT with 150 mW/cm (0.5 and 1 mg/kg HPPH, respectively), and 29% ± 18% of the tumor was positive for Ki-67 after PDT at 75 mW/cm (1 mg/kg HPPH). Thus, the sampled nodules were representative of progressive disease. These values were slightly (but insignificantly) lower than that found in the light controls, for which 32%–35% of the section area was involved in proliferation. These decreases in proliferation with PDT are likely a consequence of treatment cytotoxicity.

### 2.3. PDT-Induced Epidermal Growth Factor Receptor (EGFR) Signaling

**Figure 4:**
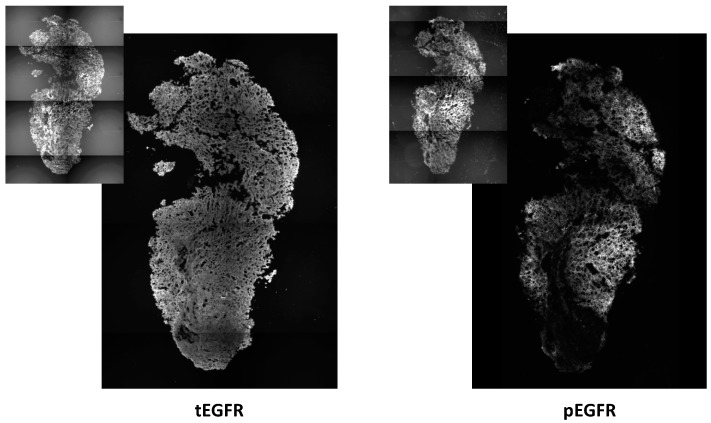
Epidermal growth factor receptor (EGFR) expression and activation is detected in tumor nodules with an incomplete response to intrathoracic PDT. Levels of total EGFR (tEGFR) and phosphorylated EGFR (pEGFR) were identified by immunohistochemistry. Insets depict fluorescence of red fluorescence protein (RFP) that identifies tumor cells. Disease was identified by bioluminescence imaging after intrathoracic PDT (treatment at 150 mW/cm, 1 mg/kg HPPH). Images represent an area of 1.7 mm × 2.6 mm (photographed at 10× magnification).

For the study of survival signaling, tumor with an incomplete response to PDT was identified by bioluminescence imaging and excised on day 16, four days after PDT. Total and activated EGFR was studied in these tumors by immunohistochemical staining and image analysis. PDT-treated disease demonstrated expression of EGFR throughout the nodule. Overall, strong staining for both total EGFR (tEGFR) and phosphorylated EGFR (pEGFR) was detected over widespread areas (Figure 4). EGFR expression was predominantly associated with tumor cells, as shown by the high correspondence between spatial distributions of tEGFR or pEGFR and the RFP fluorescent signal (insets of Figure 4) that identified tumor cells within the image. PDT did not change tEGFR expression and mean levels of tEGFR staining intensity were similar among all of the tested PDT and control conditions. Neither were levels of pEGFR affected by treatment. However, in regard to pEGFR expression, we note that variation in staining intensities was visible throughout a tumor. The expression of EGFR has previously been reported in xenografts of H460 tumors prior to therapeutic intervention [27], which agrees well with our detection of its expression and activation in control disease.

### 2.4. EGFR Activation in Proliferating Tissue

**Figure 5:**
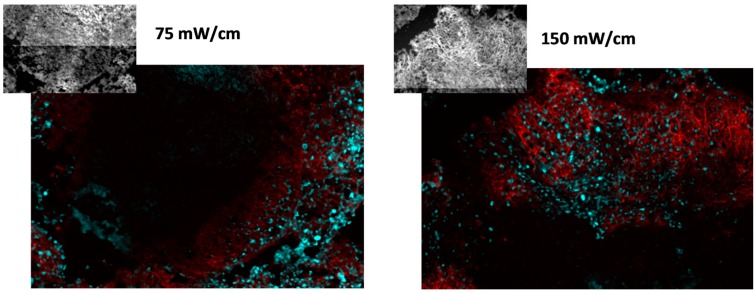
EFGR activation is associated with proliferating cells. Proliferation is identified by Ki-67 (cyan) and EGFR activation by phosphorylated EGFR (pEGFR; red). Tumor cells are identified by RFP, shown in the insets. Images represent an area of 0.9 mm × 0.6 mm (photographed at 10× magnification). PDT was delivered at 75 or 150 mW/cm, 1 mg/kg HPPH.

To more specifically consider EGFR activation in association with the proliferating areas in PDT-treated tumors, pEGFR expression was determined in exclusively the cellular areas that stained positive for Ki-67. Investigations were conducted using tumor sections that were co-labeled for pEGFR and Ki-67, and analyses determined the level of pEGFR expression in tissue that co-stained for Ki-67. Figure 5 depicts representative images of Ki-67 (cyan) and pEGFR (red) staining in nodules from mice treated at 75 or 150 mW/cm. The insets depict fluorescence from RFP that indicates the location of tumor cells within the images. Distinct regions of proliferating cells that associated with pEGFR expression were visible for both treatment conditions.

**Figure 6:**
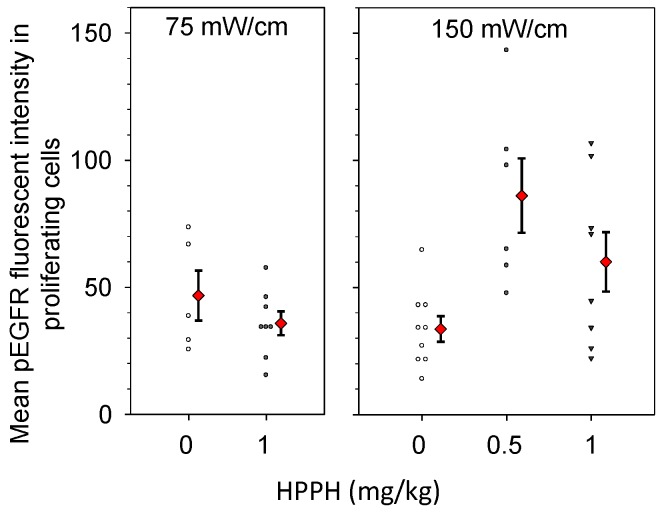
PDT increases EGFR activation in the proliferating areas of intrathoracic tumors treated with a fluence rate of 150 mW/cm. Immunohistochemistry was used to quantify levels of phosphorylated EGFR (pEGFR) in areas that were positive for proliferation by Ki-67 staining. Controls received no photosensitizer (0 mg/kg HPPH), but did receive illumination at the fluence rate that corresponded to the PDT condition. Treatment of the tumor-bearing thoracic cavity was to a total dose of 50 J/cm. Open symbols (controls) and closed black symbols (treatment groups) indicate the pEGFR staining intensity in individual mice. Red diamonds indicate the mean and standard error (error bars) for each group.

Intensity levels for pEGFR expression in Ki67-positive cells were quantified from images such as those shown in Figure 5, and the resulting data are plotted in Figure 6. Controls consisted of animals that received only light at the same fluence rate (a photosensitizer dose of 0 mg/kg). We conducted comparisons to a control condition of light-alone because our previous work had shown that high fluence rate illumination, even in the absence of photosensitizer, can lead to histological evidence of minor tissue damage in the thoracic cavity [24]. Nevertheless, pEGFR staining levels were similar among controls conditions for drug without light and light without drug. Among these controls, mean fluorescent intensity levels ranged from 33 to 46, which is essentially the full range of values represented by the light controls (see Figure 6). We found that treatment at the lower incident fluence rate of 75 mW/cm did not increase pEGFR expression in the Ki-67 positive tissue relative to pEGFR expression in control disease. In contrast, both treatments at 150 mW/cm resulted in higher mean pEGFR expression in the proliferating areas of treated tumor. At 150 mW/cm, with a drug dose of 0.5 mg/kg, the pEGFR staining level was 2.08 times [95% confidence interval (CI) 1.03, 4.20] greater than controls (*p* = 0.043), and with a drug dose of 1 mg/kg the pEGFR staining levels were 1.70 times (95% CI 0.87, 3.32) greater than control values. Notably, both control illuminations in the absence of HPPH (0 mg/kg HPPH) produced similar levels of EGFR activation so treatment with light alone was not associated with fluence rate effects.

### 2.5. Discussion

From the above studies, we conclude that a treatment condition of 75 mW/cm (1 mg/kg of HPPH) was effective at reducing tumor burden in intrathoracic PDT, but not associated with increased EGFR activation in regions of proliferating tumor cells after PDT. In contrast, PDT with 150 mW/cm (at either 0.5 or 1 mg/kg HPPH) led to overall modest decreases in tumor burden and increases in EGFR activation in proliferating tumor post-PDT. From these findings we make several observations on the relevance of fluence rate in intracavitory PDT.

Firstly, these data suggest that lower fluence rates may be more effective than high rates in reducing intracavitory tumor burden. This is consistent with much data in other tumor models that support the use of lower fluence rate to increase PDT efficacy [25,28,29,30]. Intracavitory PDT as performed in the thoracic and peritoneal cavities is accompanied by tissue exposure to a broad range of fluence rates, but shifting to a lower incident fluence rate will correspondingly lower this range. Thus, low fluence rate is expected to be similarly beneficial for intracavitory PDT as it is for other illumination geometries. Indeed, Rizvi *et al.* [31] have reported that PDT with lower fluence rates is more effective in treatment of the mouse peritoneal cavity. Their study of PDT for diffuse murine ovarian cancer used photoimmunoconjugates of the photosensitizer chlorin e6 with the EGFR-targeting antibody Erbitux in conjunction with intraperitoneal illumination. This differed from our study in that the photoimmunoconjugate served to target PDT to the EGFR receptor. Nevertheless, as we found with untargeted HPPH as a photosensitizer, residual tumor weight was lower in animals that received lower fluence rate. The cytotoxic benefit of lower fluence rate is typically attributed to better maintenance of tumor oxygenation, leading to more direct cellular damage from PDT [9]. Low fluence rate at sub-curative light doses can also promote an inflammatory response that contributes to tumor control [11]. There is evidence of PDT-induced inflammation in the present study. However, it is unlikely that an inflammatory response is the sole factor accounting for the better response at low fluence rate, especially if its effects are mediated through anti-tumor immunity because the present study used nude animals and a short followup (4 days post-PDT). Low fluence rate is also associated with more vascular damage in the post-PDT time course [32]. However, in the present study, we expect that fluence rate differentials in vascular effects were small because we did not find lower fluence rate to increase morbidity (data not shown). In the context of treating the entire thoracic cavity, one would expect morbidity to accompany vascular damage so it seems likely that fluence rate alteration of vascular effects was minimal in this study.

A second observation of our studies is that in the proliferating regions of post-treatment disease, the activation of survival factors, such as EGFR, may be less prominent when illumination is delivered at lower fluence rate. Less proliferation after lower fluence rate treatment could also contribute to a smaller tumor burden after PDT at 75 mW/cm. Our studies did not test fluence rate effects on proliferation *per se* because we intentionally selected proliferating disease for the immunohistochemistry analyses. These analyses focused on the study of EGFR, and the study design allowed for the evaluation of EGFR in the specific tissue that it is most expected to affect, *i.e.*, the recurring tumor post-therapy.

Increased expression and/or activation of proliferation-associated signaling, such as that of the EGFR pathway, is associated with NSCLC and other types of cancer [33]. However, divergent data exist in regard to PDT effect on EGFR expression and activation. Some have found PDT to decrease the expression of EGFR in tumors *in vivo* or cells *in vitro*, while others have noted PDT-induced increases in both EGFR levels and its activation [14,21,34,35,36]. Bhuvaneswari *et al.* [37] reported that hypericin-mediated PDT of xenografts of bladder tumors led to small increases in EGFR expression as measured by Western blotting, and more recently a similar finding was published for EGFR detection by immunohistochemistry after chlorin e6-mediated PDT of xenografts of oral squamous cell carcinoma [21]. Edmonds *et al.* [34] reported PDT with benzoporphyrin derivative of multiple cancer cell lines to increase EGFR activation in the absence of changes in total EGFR levels. In general, studies of EGFR expression after PDT have spanned a range of cell types, photosensitizers, and illumination conditions, which may account for heterogeneity in responses. For example, in considering the photosensitizer, the potential for a drug to localize to the plasma membrane *versus* the cytosolic organelles of a cell could play a role in PDT-mediated damage of the EGFR under some [35], but not all conditions [38]. In regard to fluence rate, very little is known about its effect on the activation of survival signaling, including molecules in the EGFR pathway. In the present manuscript we have uniquely investigated fluence rate effects on EGFR signaling *in vivo*.

The mechanisms behind a fluence rate effect on EGFR activation could be related to fluence rate-dependent PDT cytotoxicity. Greater cytotoxicity at lower fluence rates could increase photochemical damage to cells and cell surface receptors, thereby reducing the potential for PDT-induced increases in survival signaling. However, in the case of our work we note that analyses of EGFR were focused on proliferating tumor cells of disease with an incomplete response. We intentionally selected for disease with minimal photochemical damage in order to understand the role of EGFR expression/activation in disease progression. In *in vitro* studies of sub-lethal PDT, others have noted that EGFR mRNA and protein levels were lower in the PDT-derived variants of repeated illumination compared to the parental cell lines, and this was accompanied by a reduced invasive potential [39]. This suggests that the daughters of PDT-treated cells may downregulate EGFR expression. In contrast to these results, an *in vivo* study on the molecular effects of sub-lethal PDT found that PDT of brain led to increases in EGFR activation [40]. These studies were performed at doses that were insufficient to produce tissue damage and there was no change in total EGFR levels, as we found in the present study. Thus, it seems that changes in EGFR activation may occur independent of cytotoxicity.

Tumor microenvironment could play a role in EGFR activation. Higher fluence rates are well established to contribute to the development of hypoxia during PDT and EGFR is activated under hypoxic conditions. An association between PDT-created hypoxia and EGFR expression was studied by Zheng *et al.* [40], in the above mentioned study of sub-lethal PDT to the murine brain. Sub-lethal Photofrin-PDT of the murine brain increased the expression of hypoxia-responsive molecules, including the transcription factor hypoxia-inducible factor 1-α (HIF1α) and a disintegrin and metalloprotease domain 17 (ADAM17), an enzyme with a role in hypoxia-associated tumor invasion. The activation of EGFR was increased, as well as that of AKT, a downstream signaling molecule that regulates apoptosis and cellular proliferation and migration. These data support a role for hypoxia in the activation of EGFR in sub-lethally damaged tissue. Yet, it is worth noting that these studies (as well as those of the present manuscript) were performed at timepoints of several days after PDT. Any effect of microenvironment on EGFR signaling will accordingly incorporate the conditions of the post-PDT microenvironment, in addition to that present during illumination itself.

Collectively, the data of this study demonstrate a lower intrathoracic fluence rate of 75 mW/cm to increase PDT efficacy (reduce tumor burden) and decrease EGFR activation in the proliferating cells of post-treatment disease. It is reported that lower fluence rate can increase PDT cytotoxicity both through direct (photooxidative) and indirect (e.g., immune/inflammatory reactions at sub-curative light doses) mechanisms, which may contribute to its increased effectiveness in our study. Moreover, we show that there is weaker activation of EGFR signaling in the proliferative areas of tumor treated with low fluence rate. This could also contribute to the smaller tumor burden after PDT at 75 mW/cm.

## 3. Experimental Section

### 3.1. Cell Line and Tumor Propagation

For the purposes of *in vivo* imaging by bioluminescence and fluorescence microscopy by RFP, H460 large cell lung cancer cells (ATCC, Manassas, VA, USA) were transfected with a triple reporter plasmid (hrl-mrfp-ttk) that was kindly provided by Dr. Gambhir (Stanford University School of Medicine, Stanford, CA, USA). Transfection was performed by electroporation and RFP positive cells were isolated by flow cytometry. Cells were maintained in RPMI-1640 medium (ATCC) supplemented with 10% fetal bovine serum (Gibco, Carlsbad, CA, USA), 2 mM l-glutamine (Gibco), 100 units/mL penicillin (Gibco), and 100 mg/mL streptomycin (Gibco) in a humidified atmosphere with 5% CO_2_ at 37 °C. For intrathoracic tumor propagation, cells were suspended at 1 × 10^6^ cells/mL in a 1:1 solution of phenol red-free Matrigel Basement Membrane Matrix (BD Biosciences, San Jose, CA, USA) and normal saline. Athymic Ncr-nu/nu female mice (NCI-Frederick, Frederick, MD, USA; Taconic, Hudson, NY, USA) of 7–9 weeks in age were anesthetized with ketamine/xylazine and percutaneously injected with 50 µL of the cell suspension. The needle was inserted through the intercostal muscle into the right lateral thorax at the dorsal axillary line approximately one centimeter above the caudal edge of the ribcage, as has been previously described [24]. Animals were under the care of the University of Pennsylvania Laboratory Animal Resources. All studies were approved by the University of Pennsylvania Institutional Animal Care and Use Committee (Protocol number 803526; approval 26 March 2011).

### 3.2. Photodynamic Therapy

Based on previously published growth curves for intrathoracic burden of H460 tumor [24], PDT was performed at 12 days after inoculation of the tumor cells. HPPH (Photochlor^®^; 2-(1-hexyloxyethyl)-2-devinyl pyropheophorbide-a), kindly provided by B.W. Henderson and T.J. Dougherty (Roswell Park Cancer Institute, Buffalo, NY, USA), was used as the photosensitizer. The dry powder of HPPH was formulated as a stock solution (~500 μg/mL) in 5% dextrose (in water) with 2% ethyl alcohol (95%), and 1% polysorbate 80; adjusted to pH 7.3–7.5 using 0.1 M sodium carbonate. The drug was diluted to a final concentration of 0.1 mg/mL in D5W and mice received a dose of 0.5 or 1 mg/kg HPPH (via tail vein). At ~24 h after HPPH injection, interstitial illumination of the thoracic cavity was performed using a customized cylindrical diffusing fiber (active length of 1 cm; diameter 200 μm; Rare Earth Medical, Inc. West Yarmouth, MA, USA). The cylindrical diffusing fiber was positioned through the bore of a 19-gauge needle that had been inserted ~1.2 cm into the right side of the thoracic cavity as described for tumor cell inoculation. The needle was withdrawn by sliding it along the external portion of the fiber, leaving the 1-cm active length of the fiber within the cavity. Illumination was performed using a 661-nm diode laser (B & W Tek, Inc. Newark, DE, USA) at a fluence rate of either 75 or 150 mW/cm (measured output from the fiber) and a fluence of 50 J/cm. An integrating sphere (Diomed, Inc., Andover, MA, USA) was used to measure power output from the fiber. During the delivery of PDT, animals were anesthetized using isoflurane (in medical air; VetEquip anesthesia machine, Pleasanton, CA, USA). Tumor response to PDT was determined by dissection and weighing of tumor burden in animals euthanized at four days after treatment. Tumor weight was compared among control (no light or drug, drug-alone, light-alone) animals and those treated at each fluence rate.

### 3.3. Optical Imaging

Anesthetized (isoflurane) animals were imaged by a Perkin Elmer IVIS Lumina II (PerkinElmer, Hopkinton, MA, USA). Each mouse was injected intra-peritoneally (i.p.) with 150 mg/kg Luciferin in saline, and imaging commenced 10–15 min later with the animals in the prone position. Images were collected at intervals of ~5–7 min in order to detect the peak signal. Longitudinal monitoring of intrathoracic tumor burden was performed, with each animal imaged on post-tumoring day 8, 10, 12 (day of PDT), 14, and 16. On the last day of imaging (day 16), two or three luciferase positive tumor nodules were selected in each animal based on a well circumscribed appearance and continued growth after PDT (see analyses described below). These were excised and frozen for immunohistochemistry from a total of three mice per treatment condition.

Visualization and quantification of bioluminescent images were performed using Living Image software (PerkinElmer). Regions of interest (ROI) were manually drawn around well-circumscribed bioluminescent hotspots and used to monitor changes in signal over the imaging timecourse. Luminescent signal is reported as the radiance, in units of photons/s/cm^2^/steradian, as calculated by the software. Average radiance is a metric that divides total bioluminescent flux by the size of the ROI.

### 3.4. Immunohistochemistry

Cell proliferation, Gr-1, total EGFR (tEGFR) and phosphorylated EGFR (pEGFR) were identified by staining of frozen sections with antibodies against Ki-67 (BD Pharmingen, San Diego, CA, USA), Gr-1 (Biolegend, San Diego, CA, USA), EGFR/ErbB1 (R & D Systems, Minneapolis, MN, USA) and phospho-EGFR (Y1068; Abcam, Cambridge, MA, USA), respectively. Excised tumor nodules were fixed in 1% paraformaldehyde (1 h at 4 °C), rinsed in phosphate buffered saline and sucrose, and frozen in OCT. Sections (14 μm) were cut using a cryostat and allowed to dry for 1 h at room temperature followed by rehydration in 1× PBS for 10 min. Staining (1 h) was performed for Ki-67 (1:100) and pEGFR (1:25) in the same section, while total tEGFR (1:50) and Gr-1 (1:100) were evaluated in adjacent sections. Respective to the Ki-67, Gr-1, tEGFR and pEGFR antibodies, secondary antibodies of Cy5-conjugated rat anti-mouse (1:100 for 1 h; Jackson Immunoresearch, West Grove, PA, USA), Cy5-conjugated mouse anti-rat (1:50 for 1 h; Jackson Immunoresearch), fluorescein isothiocyanate (FITC)-conjugated rabbit anti-goat, and FITC-conjugated donkey anti-rabbit (both of the latter at 1:25 for 1 h; Jackson Immunoresearch) were used. During photography, Hoechst 33342 (20 μM) was used to label tissue and compared to RFP to confirm the presence of tumor. Tiled images of each tumor nodule (10×) were collected by a Nikon Eclipse 800 fluorescence microscope (Nikon Inc., Melville, NY, USA) and Photometrics Quantix CCD digital camera (Photometrics, Tucson, AZ, USA) controlled by IPLab software (Scanalytics, Inc., Fairfax, VA, USA). Analysis of Ki-67 and Gr-1 involved masking to label stained areas (Adobe Photoshop; Adobe Systems Inc., San Jose, CA, USA) followed by assessment using routines in the MATLAB Image toolbox (MathWorks, Natick, MA, USA) in order to calculate the percentage of Hoechst-identified tissue that was positive for Ki-67 or Gr-1. Phospho-EGFR and tEGFR intensity was measured in areas identified by Hoechst staining, and in the case of pEGFR, intensity values were also obtained for pEGFR that co-localized with the masks of Ki-67. Controls included slides stained with only secondary antibody, and demonstrated no staining.

### 3.5. Statistics

Data are summarized by the mean and standard deviation or standard error within each treatment group. In studies of tumor mass, Wilcoxon rank-sum (Mann–Whitney) tests were used to compare PDT at each treatment condition with its corresponding control. Analyses of immunohistochemistry results utilized mixed effects models to determine the association between pEGFR level and treatment. This approach allowed us to take into account repeated measurements (nodules) on the same mouse. Here, a log transformation was used to normalize the data. For all tests, a type I error rate was set at 0.05.

## 4. Conclusions

The studies of this manuscript investigate intrathoracic PDT at incident fluence rates of 75 and 150 mW/cm in order to delineate whether incident fluence rate can play a role in PDT outcome for illumination of complex geometries such as the thoracic cavity. Compared to illumination at 150 mW/cm, PDT at 75 mW/cm produced a greater reduction in tumor burden. This may in part be attributed to an activation of EGFR signaling in post-treatment proliferating disease when illumination was given at the higher rate of 150 mW/cm. EGFR is well established to contribute to resistance to cancer therapies and molecular targeting of the EGFR pathway can improve therapeutic outcomes to PDT and other types of treatment [20,21,37,41]. These data suggest that the addition of EGFR-targeting therapy may be particularly beneficial in combination with intrathoracic PDT when higher fluence rates are employed. Given the complexities of intrathoracic illumination, it is likely that these results are also relevant to other more simple illumination geometries.
